# 1089. Use of a Whole-Body Quantitative System Pharmacology Physiologically-Based Pharmacokinetic (QSP/PBPK) Model to Support Dose Selection of ADG20: an Extended Half-Life Monoclonal Antibody Being Developed for the Prevention of Coronavirus Disease (COVID-19)

**DOI:** 10.1093/ofid/ofab466.1283

**Published:** 2021-12-04

**Authors:** Scott A Van Wart, Evan D Tarbell, Kristin Narayan, Laura M Walker, Lynn E Connolly, Paul G Ambrose

**Affiliations:** 1 Enhanced Pharmacodynamics LLC, Buffalo, New York; 2 Adagio Therapeutics, Inc., Waltham, Massachusetts; 3 Adimab LLC, Lebanon, New Hampshire

## Abstract

**Background:**

ADG20 is a fully human IgG1 monoclonal antibody engineered to have potent and broad neutralization against severe acute respiratory syndrome coronavirus 2 (SARS-CoV-2) and other SARS-like CoVs with pandemic potential as well as an extended-half-life. ADG20 is administered intramuscularly (IM). A QSP/PBPK model was constructed to support dose selection for a COVID-19 Phase 2/3 prevention trial (EVADE: NCT04859517).

**Methods:**

A QSP/PBPK model and a CDC reference adult body weight distribution (45–150 kg) were used to simulate 1000 concentration-time profiles for candidate single-dose regimens of ADG20 (150–450 mg IM). As serum virus neutralizing antibody (sVNA) titers are reportedly a key correlate of protection from COVID-19, a regression equation between time-matched serum ADG20 concentrations (following a 300 mg IM dose) and sVNA titers was developed using measured titers against authentic SARS-CoV-2 determined by a plaque reduction neutralization assay. Projected ADG20 serum concentrations relative to neutralization potency in vitro (90% inhibitory concentration [IC_90_]) for authentic SARS-CoV-2 were also evaluated.

**Results:**

The measured 50% neutralization titer (MN50; geometric mean [coefficient of variation, %]) was 1382 (32.7%) 13 days after a single 300 mg IM dose of ADG20. This was within the range of peak sVNA titers reported for COVID-19 vaccine recipients. Using the linear equation relating serum ADG20 concentration to time matched individual MN50 titers and the QSP/PBPK median PK prediction, the anticipated median MN50 exceeded the threshold for protection from SARS-CoV-2 infection established in a non-human primate adoptive transfer model for up to 52 weeks. Based on the QSP/PBPK median PK prediction, median ADG20 serum concentrations are projected to remain >100-fold above the ADG20 IC_90_ value of 0.011 mg/L against authentic SARS-CoV-2 for up to 52 weeks (Figure).

**Conclusion:**

Following administration of a single 300 mg IM dose, sVNA titers and concentrations of ADG20 are projected to remain above thresholds anticipated to be required for protection against COVID-19 for up to 52 weeks. These data support the evaluation of a single ADG20 300 mg IM dose for the prevention of COVID-19.

Figure. QSP/PBPK model forecast of ADG20 300 mg IM in adults.

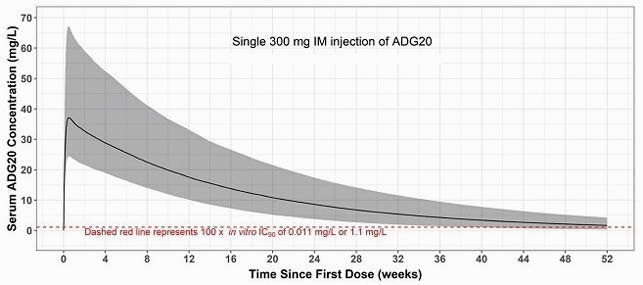

Predicted median serum ADG20 concentration is shown with the dotted line representing 100× in vitro IC90 of 0.011 mg/L or 1.1 mg/L; the solid black line represents the simulated median; the shaded area represents the 90% prediction interval. The predicted median half-life of ADG20 300 mg IM exceeded 74 days. PBPK model inputs include Ln-normal Kd,FcRn of 9.55 nM (10% IIV); IM bioavailability of 100%; 15% IIV on muscle lymph RC; and Centers for Disease Control and Prevention weight distribution of 45–150 kg. FcRn, neonatal Fc receptor; IIV, inter-individual variability; Kd, dissociation constant; Ln, log-normal; RC, reflection coefficient.

**Disclosures:**

**Scott A. Van Wart, PhD**, **Adagio Therapeutics, Inc.** (Independent Contractor) **Evan D. Tarbell, PhD**, **Adagio Therapeutics, Inc.** (Independent Contractor) **Kristin Narayan, PhD**, **Adagio Therapeutics, Inc.** (Employee) **Laura M. Walker, PhD**, **Adagio Therapeutics, Inc.** (Other Financial or Material Support, Laura M. Walker is an inventor on a patent application submitted by Adagio Therapeutics, Inc., describing the engineered SARS-CoV-2 antibody.) **Lynn E. Connolly, MD, PhD**, **Adagio Therapeutics, Inc.** (Employee) **Paul G. Ambrose, PharmD**, **Adagio Therapeutics, Inc.** (Employee)

